# Supplementary material for: A Novel DNA Variant in SMARCA4 Gene Found in a Patient Affected by Early Onset Colon Cancer
Source: Int J Mol Sci. 2024 Feb 27;25(5):2716. doi: 10.3390/ijms25052716 (PMC10932324; doi:10.3390/ijms25052716)
Supplement: Supplementary file 1 [file ijms-25-02716-s001.zip › ijms-2892934-supplementary.pdf]

International Journal of Molecular Sciences

MDPI

Supplementary Materials

A Novel DNA Variant in *SMARCA4* Gene Found in a Patient Affected by Early Onset Colon Cancer

Federica Di Maggio [1,2,†], Giuseppe Boccia [3,†], Marcella Nunziato [1,2], Marcello Filotico [3], Vincenzo Montesarchio [4], Maria D'Armiento [5], Francesco Corcione [3,*] and Francesco Salvatore [1,2,*]

1 CEINGE-Biotecnologie Avanzate Franco Salvatore, 80145 Naples, Italy; dimaggio@ceinge.unina.it (F.D.M.); nunziato@ceinge.unina.it (M.N.)
2 Department of Molecular Medicine and Medical Biotechnologies, University of Naples "Federico II", 80131 Naples, Italy
3 Department of Public Health, University of Naples "Federico II", 80131 Naples, Italy; giuseppe.boccia@unina.it (G.B.); dottmarcellofilotico@gmail.com (M.F.)
4 Division of Medical Oncology, AORN dei Colli-Monaldi Hospital, 80131 Naples, Italy; vincenzo.montesarchio@ospedalideicolli.it
5 Pathology Unit, Department of Public Health, University of Naples "Federico II", 80131 Naples, Italy; maria.darmiento@unina.it
* Correspondence: francesco.corcione@clinicamediterranea.it (F.C.); salvator@unina.it (F.S.)
† These authors contributed equally to this work.

Supplementary Table S1. Composition of Advanced +++ medium

Product	Catalogue Number	Product Concentration
Advanced DMEM/F-12	12634010	up to 50 mL
Primocin	ant-pm-2	50 mg/ml
Hepes (1M)	15630-080	100x
Glutamax	35050061	100x

Supplementary Table S2. Composition of Cell culture medium

Reagent	*Concentration*	*Catalogue Number*
A83-01	0,025 mM	2939
mEGF	0,05 µg/ml	PMG8043
Gastrin	0,1 µM	1003377
mNoggin	0,1 µg/mL	250-38
IGF-I	0,1 µg/mL	100-11
N-acetylcysteine	1,25 mM	A9165
Nicotinamide	10 mM	N0636-100G
B27 supplement	1 µl/mL	17504044
Advanced +++	up to 50 mL	
